# Proficiency testing of diagnosis in histopathology and immunohistochemistry of breast pathology in China: results from a pilot work of National Single Disease Quality Control Program for breast cancer

**DOI:** 10.1186/s12885-023-11777-3

**Published:** 2024-01-02

**Authors:** Xuemin Xue, Lei Guo, Changyuan Guo, Lin Li, Lin Yang, Xin Wang, Wei Rao, Pei Yuan, Jiali Mu, Jiangtao Li, Bingning Wang, Quan Zhou, Wentao Yang, Yueping Liu, Weicheng Xue, Rujing Jia, Wenjing Yang, Jianming Ying

**Affiliations:** 1 Department of Pathology, National Cancer Center/National Clinical Research Center for Cancer/Cancer Hospital, Chinese Academy of Medical Sciences and Peking Union Medical College, 17 Panjiayuan Nanli, Chaoyang District, Beijing, 100021 China; 2 Department of Pathology, Fudan University Shanghai Cancer Center, Shanghai, 200032 China; 3 Department of Oncology, Shanghai Medical College, Fudan University, Shanghai, 200032 China; 4 Department of Pathology, The Fourth Hospital of Hebei Medical University, Shijiazhuang, 050011 China; 5 Key Laboratory of Carcinogenesis and Translational Research (Ministry of Education), Department of Pathology, Peking University Cancer Hospital & Institute, Beijing, 100142 China; 6 Special Standard Laboratory Accreditation Department, National Accreditation Service for Conformity Assessment, 8 Nanhuashi Street, Dongcheng District, Beijing, 100062 China; 7 Office for Cancer Diagnosis and Treatment Quality Control, National Cancer Center/National Clinical Research Center for Cancer/Cancer Hospital, Chinese Academy of Medical Sciences and Peking Union Medical College, 17 Panjiayuan Nanli, Chaoyang District, Beijing, 100021 China

**Keywords:** Breast pathology, Proficiency testing, Quality control, Accuracy, Histopathology, Immunohistochemistry

## Abstract

**Aim:**

Pathologists are currently supposed to be aware of both domestic and international guidelines for breast cancer diagnosis, but it is unclear how successfully these guidelines have been integrated into routine clinical practice in China. Thus, this national proficiency testing (PT) scheme for breast pathology was set up to conduct a baseline assessment of the diagnostic capability of pathologists in China.

**Methods:**

This national PT plan is designed and implemented according to the “Conformity assessment—General requirements for proficiency testing” (GB/T27043—2012/ISO/IEC 17043:2010). Five cases of breast cancer with six key items, including histologic type, grade, ER, PR, HER2, and Ki67, were selected for testing among 96 participants. The final PT results were published on the website of the National Quality Control Center for Cancer (http://117.133.40.88:3927/cn/col22/362).

**Results:**

Our study demonstrated that the median PT score was 89.5 (54–100). Two institutions with scores < 67 were deemed unacceptable. The accuracy of histologic type, ER, PR, HER2, and Ki67 was satisfactory (all > 86%). However, the histologic grade showed low accuracy (74.0%). The unacceptable results mainly included incorrect evaluation of histologic grade (36.7%), inaccurate evaluation of ER/PR/HER2/Ki67 (28.2%), incorrect identification of C-AD as IBC-NST (15.7%), inappropriate use of 1+/2+/3+ rather than staining percentage for ER/PR (6.1%), misclassification of ER/PR < 1% weak expression as positive staining (1.4%), and no evaluation of histologic grade in ILC, MC, and IMC (5.8%).

**Conclusions:**

our nationwide PT program exhibited a satisfactory baseline assessment of the diagnostic capability of pathologists in China. More importantly, we identify some areas for further improvement.

## Introduction

Breast cancer in females is currently the leading cause of cancer incidence and the fifth leading cause of cancer mortality worldwide in 2020, according to the GLOBOCAN 2020 report [1]. The Chinese population-based cancer registry data in 2015 also exhibited breast cancer with a significant increase in age-standardized incidence rate by the world standard population [2]. Moreover, breast cancer survival rates in China are lower than those in the United States [3]. Therefore, it is necessary for China to make further efforts to provide more effective cancer care for breast cancer patients.

Concurrently, one of the ongoing efforts to enhance breast cancer care in China is the National Single Disease Quality Control Program (NSDQCP) for tumors, which has been implemented by the National Cancer Center/National Cancer Quality Control Center (NCC/NCQCC) since 2019. In this program, breast cancer was selected as the first tumor type to carry out the pilot work, aiming to promote inter-institutional uniformity and standardization of diagnosis and treatment, and to improve the long-term survival rate and quality of life of patients. To achieve these goals, the quality of pathology reporting must be viewed as an intrinsic component of breast cancer care.

So far, Europe and the UK have highly developed international or national frameworks for external quality assessment (EQA) in the diagnosis of breast pathology [4, 5]. However, there was no similar nationwide program to assess the diagnostic proficiency in China. Generally, pathologists are supposed to be aware of both domestic and international guidelines for breast cancer diagnosis; however, it is unclear how successfully these guidelines have been integrated into routine clinical practice in China. Therefore, in 2022, the NCC/NCQCC and China National Accreditation Service for Conformity Assessment (CNAS) jointly organized the National Proficiency Testing (PT) for the Pathological Diagnostic Capability of Breast Cancer. This PT program is essential for conducting a baseline assessment of diagnostic capability in China and identifying problems for future improvement. Our present paper here summarizes the findings and experiences of this national PT scheme.

## Materials and methods

### Program design

This nationwide PT program for pathological diagnosis in China was designed and implemented jointly by the NCC/NQCCC and CNAS, according to the “Conformity assessment—General requirements for proficiency testing” (GB/T27043—2012/ISO/IEC 17043:2010) [6, 7]. The PT provider is the pathology department of the National Cancer Center/National Clinical Research Center for Cancer/Cancer Hospital, which has acquired certification of pathological competence through CNAS against the standards of ISO15189 [8]. Moreover, an expert panel of five pathologists specializing in breast pathology (Q.Z., W.Y., Y.L., W.X., and Y.J.) from various tertiary grade A hospitals was established.

### Participants

This program was piloted among the members (approximately 200) of the NSDQCP for breast cancer. Based on the registration order, the first 100 member institutions were selected to participate in this plan. All participants were required to be capable of diagnosing breast cancer and issuing clinical pathology reports independently.

### Test samples selection and preparation

For the purpose of conducting a baseline assessment in China, the most common histologic types of breast cancer according to the WHO classification [9], as suggested by the expert panel, were chose for testing, including (1) invasive breast carcinoma of no special type (IBC-NST, frequency: >70%), (2) invasive lobular carcinoma (ILC, frequency: 5–15%), (3) mucinous carcinoma (MC, frequency: 2%), (4) invasive micropapillary carcinoma (IMC, frequency: 2–0.9%), and (5) carcinoma with apocrine differentiation (C-AD, frequency: 1%).

Hematoxylin and eosin (H&E) and immunohistochemistry (IHC) glass slides of five cases of breast cancer were selected from the routine diagnostic specimen archives stored at the PT provider institution. All the specimens were fixed and processed according to the recommendations of the American Society of Clinical Oncology/College of American Pathologists (ASCP/CAP) [10, 11]. Breast cancer subtype and histologic grade were diagnosed according to the 5th World Health Organization classification of breast cancer [9]. The square or circle measure tools in the KF-Viewer software was used to help mark an identical area for mitotic counting. ER and PR testing were performed according to the ASCO/CAP guideline update (version 2020) [11]. HER2 detection was performed according to the guideline (version 2019) recommended by the breast cancer expert panel [12].

Initially, these five testing cases were chosen and reviewed by G.Y. If the quality and staining of the slides were good for testing, the corresponding glass slides, including H&E, Ecad, ER, PR, HER2, and Ki67, were scanned by J.M. using a KF-PRO-040 Magscanner system (KFBIO, China) at a magnification of 20×. The original pathological numbers on the glass slides were covered with black paper for data protection before scanning. Afterwards, these digital slides were then reviewed by the expert panel.

### Test samples distribution

Considering that all digital copies were made from the same digital sources and USB flash disks were purchased with the same model and batch, the homogeneity of the image was guaranteed. Furthermore, to prevent collusion between participants, each set of digital slides of the five cases was saved in a random order on each USB flash drive. The original pathological numbers of the five cases in each USB flash drive were replaced with common serial numbers (from 1 to 5). Meanwhile, the list of participating institutions was kept confidential throughout the program to reduce potential inter-laboratory communication.

For sample release, 100 USB flash drives were distributed simultaneously to 100 participating institutions that signed up for our national PT program. Each drive contained four files, including (1) digital slides, (2) digital slide viewer software (KF-Viewer), (3) operation guide, and (4) confirmation form for receiving status.

### Reporting system development

An online reporting system was developed for our PT program. After completing the final results on the website, each participant submitted their results online and simultaneously printed a copy of the result. This paper copy will be signed by the director of each institution and sent back to our center as a final confirmation file. Afterwards, the originally distributed USB flash disk with the signed paper copy of the result was required to be sent back to our center.

### Assigned values generation

In order to setup assigned values (gold standards) for testing, the digital slides were summitted to each expert for evaluation using the Lenovo ThinkCentre E700 with 27-inch IPS full HD Monitor. Then we collect the results from all five experts, and generated the assigned values according to the agreement among the experts. For the histologic type, histologic grade and HER2-IHC score, the results with ≥ 60% agreement were considered as the assigned values. For the ER, PR and Ki67, the median value (with the range of minimal and maximal values) of results were determined as the assigned values.

### Evaluation of results of proficiency testing

**Fig. 1:**
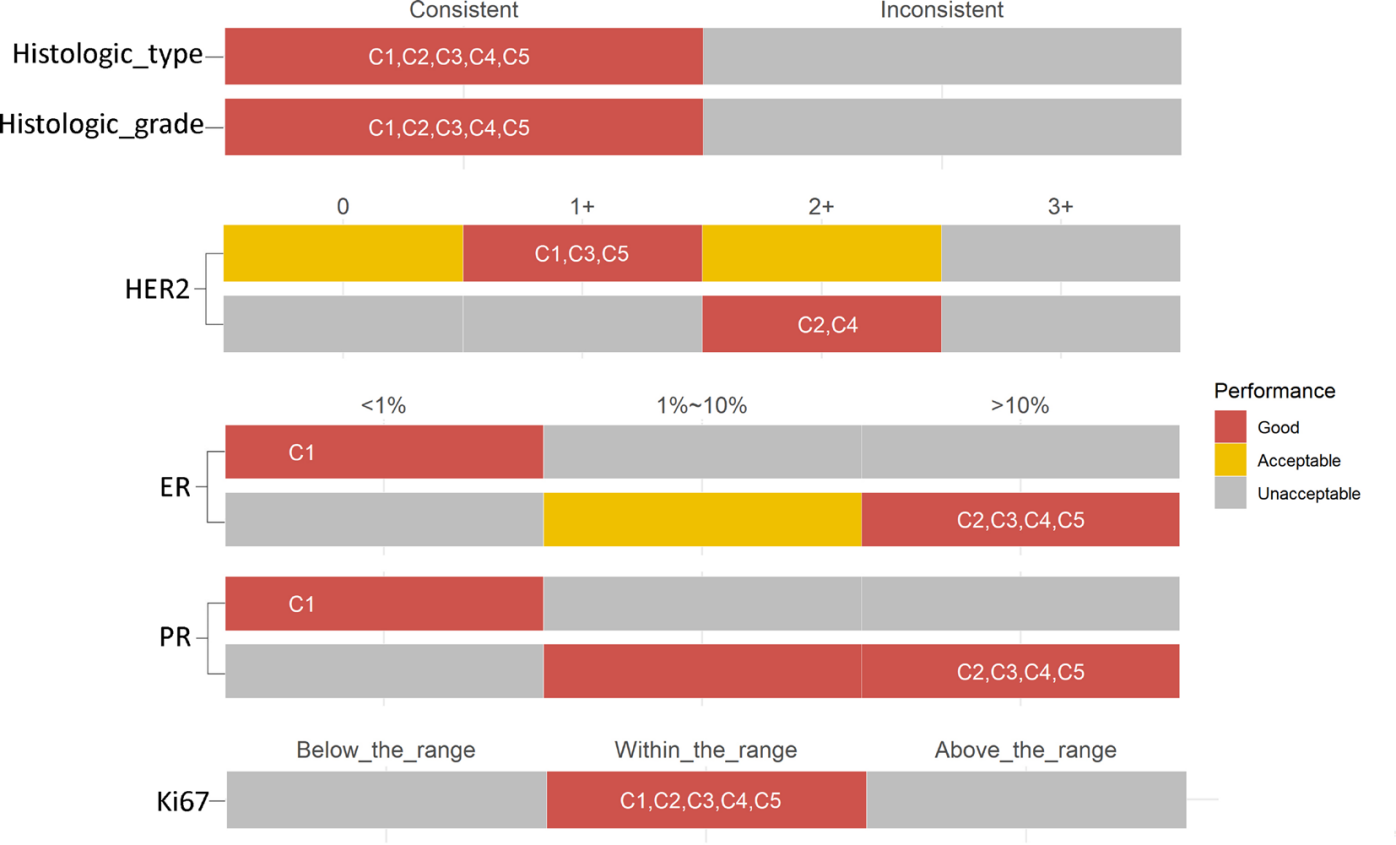
Performance evaluation system for PT results of six key items

Meanwhile, based on a full discussion and related guidelines [9–12], the assigned value and uncertainty of each item were rendered by the expert panel (Table 1). Thus, a performance evaluation system for PT was developed (Fig. 1). For example, the assigned value of HER2 for C1, C3, and C5 was 1+. Good performance was considered if some participants reported 1+ in these cases. Acceptable performance was considered if some participants reported 0 or 2+. Otherwise, unacceptable performance was determined when any 3+ was reported (Fig. 1).

**Table 1:** Assigned values of six key pathological items in five cases

No.	Type	Grade	ER	PR	HER2	Ki67
C1	C-AD	2	−, 0%	−, 0%	1+	15%
C2	IBC-NST	2	+, 80% strong	+, 60% strong	2+	15%
C3	IMC	2	+, 80% strong	+, 70% strong	1+	25%
C4	MC	1	+, 90% strong	+, 90% strong	2+	10%
C5	ILC	2	+, 80% strong	+, 20% weak	1+	30%

*C-AD* carcinoma with apocrine differentiation, *IBC-NST* invasive breast carcinoma of no special type, *IMC* invasive micropapillary carcinoma, *MC* mucinous carcinoma, *ILC* invasive lobular carcinoma

In this evaluation system, each case had 20 points: (1) 3 points for histologic type, (2) 3 points for histologic grade, (3) 4 points for HER2, (4) 4 points for ER, (5) 4 points for PR, and (6) 2 points for Ki67. A maximum PT score of 100 points was assigned to each participant.

### Data statistics

In our study, the participating institutions were expected to generally have high scores toward to the 100 points, and only a few institutions were thought to have low scores. Therefore, the PT scores should have a left-skewed distribution rather than a normal distribution. Thus, a Weibull distribution was employed to describe the probability of PT scores among participating institutions.

Herein, “fitdistr” analysis in “MASS” package to calculate the scale and shape values of Weibull distribution, and “KS.test” was used to test Weibull distribution. “ggpubr” and “REmap” packages were used to plot data.

As to the interobserver variability, intraclass correlation coefficient (ICC) was calculated for continuous variables (including ER, PR, and Ki67), and Fleiss’s Kappa (κ) was calculated for categorical data evaluated (including histologic type, histologic grade, and HER2). The levels of agreement, determined by the kappa or ICC statistic, were categorized as follows: weak (≤ 0.20), fair (0.21–0.40), moderate (0.41–0.60), good (0.61–0.80), and perfect (0.81–1.00).

All statistical analyses were performed in R (version 3.6.0, https://www.r-project.org/), and lower-tailed p < 0.01 for PT score was considered as significantly unacceptable. The final PT results were published on the website of the National Quality Control Center for Cancer (http://117.133.40.88:3927/cn/col22/362).

## Results

### Characteristics of 96 participating institutions

**Fig. 2:**
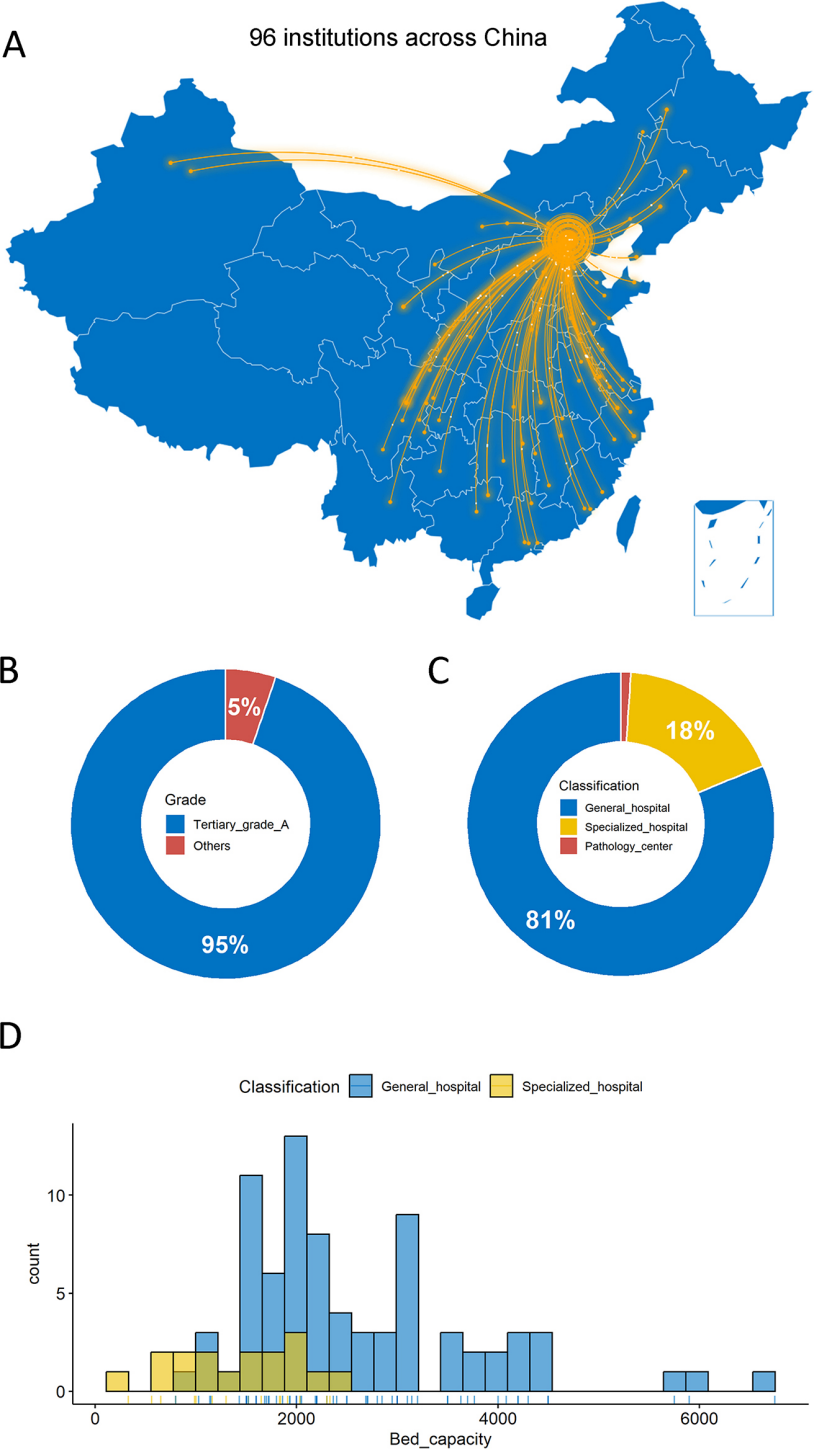
Overall characteristics of participating institutions across China. **A** Geographic distribution of 96 institutions in 26 provinces/municipalities/autonomous regions was demonstrated in the map of China. **B** Grades of 96 institutions were showed in the donut charts. **C** Classifications of 96 institutions were showed in the donut charts. **D** Bed capacity between general and specialized hospitals was showed

Initially, 100 institutions signed up to participate in this national PT program. However, we finally collected valid results from 96 institutions across 26 provinces/municipalities/autonomous regions in China (Fig. 2A). 95% of them were tertiary grade A hospitals (Fig. 2B). Meanwhile, 81% of them were general hospitals with a median bed capacity of 2200, and 18% were specialized hospitals with a median bed capacity of 1520, and the remaining one (1%) was an independent pathology center (Fig. 2C, D).

### Generation of assigned values for six items

**Fig. 3:**
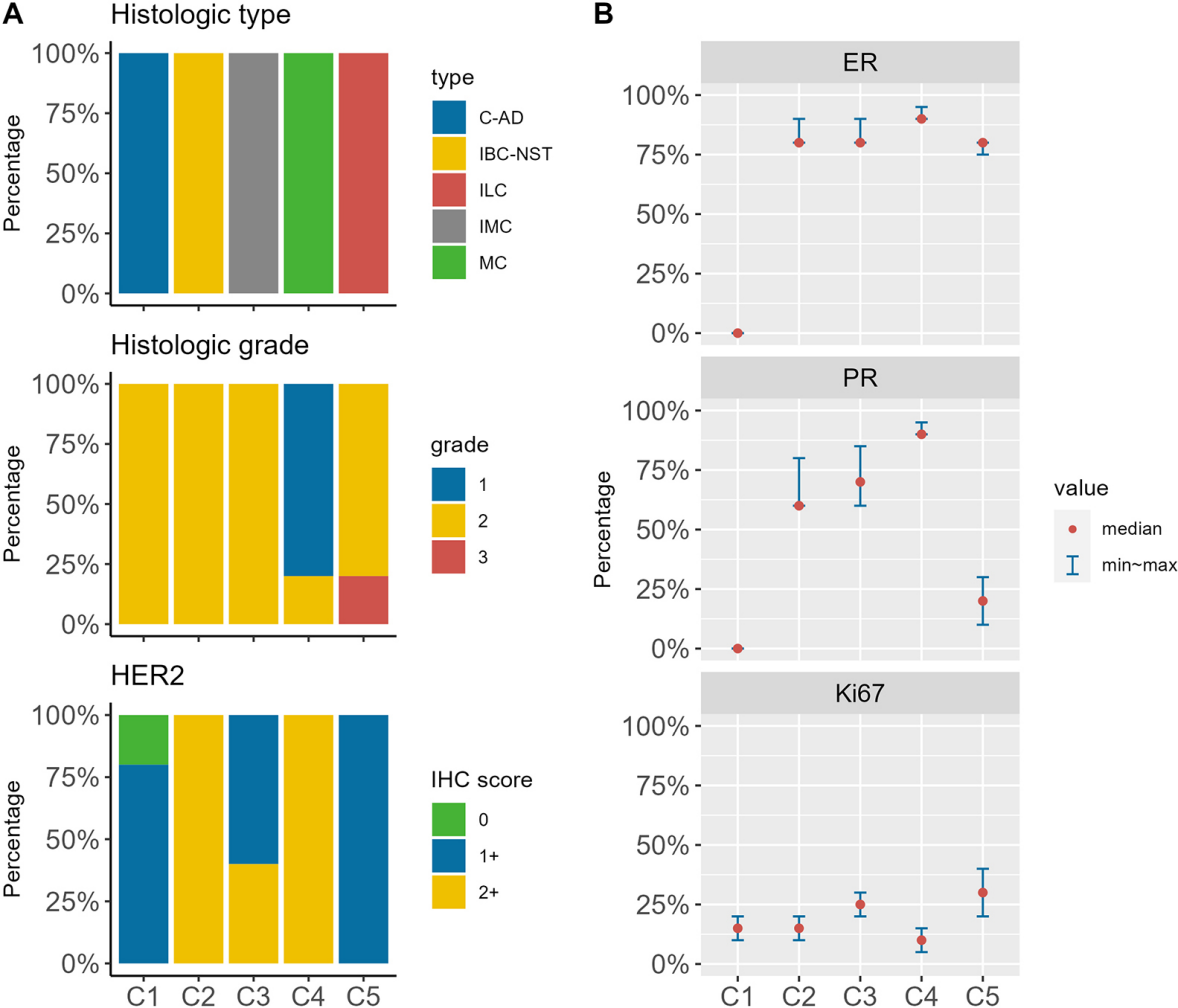
The detailed variability on the various parameters among the experts. **A** The detailed variability of histologic type, histologic grade and HER2. **B** The detailed variability of ER, PR and Ki67

The digital slides were summitted to each expert for evaluation. Afterwards, we collected the results from all five experts. The detailed variability on the various parameters among the experts was showed in the following figure (Fig. 3). Except for the HER2 score on C3 case that showed 60% agreement among the experts, the other HER2 score, histologic type and grade all demonstrated ≥ 80% agreements. Based on the agreement among the experts, the assigned value of each item was generated (Table 1).

### Performance of six key items among five cases

**Fig. 4:**
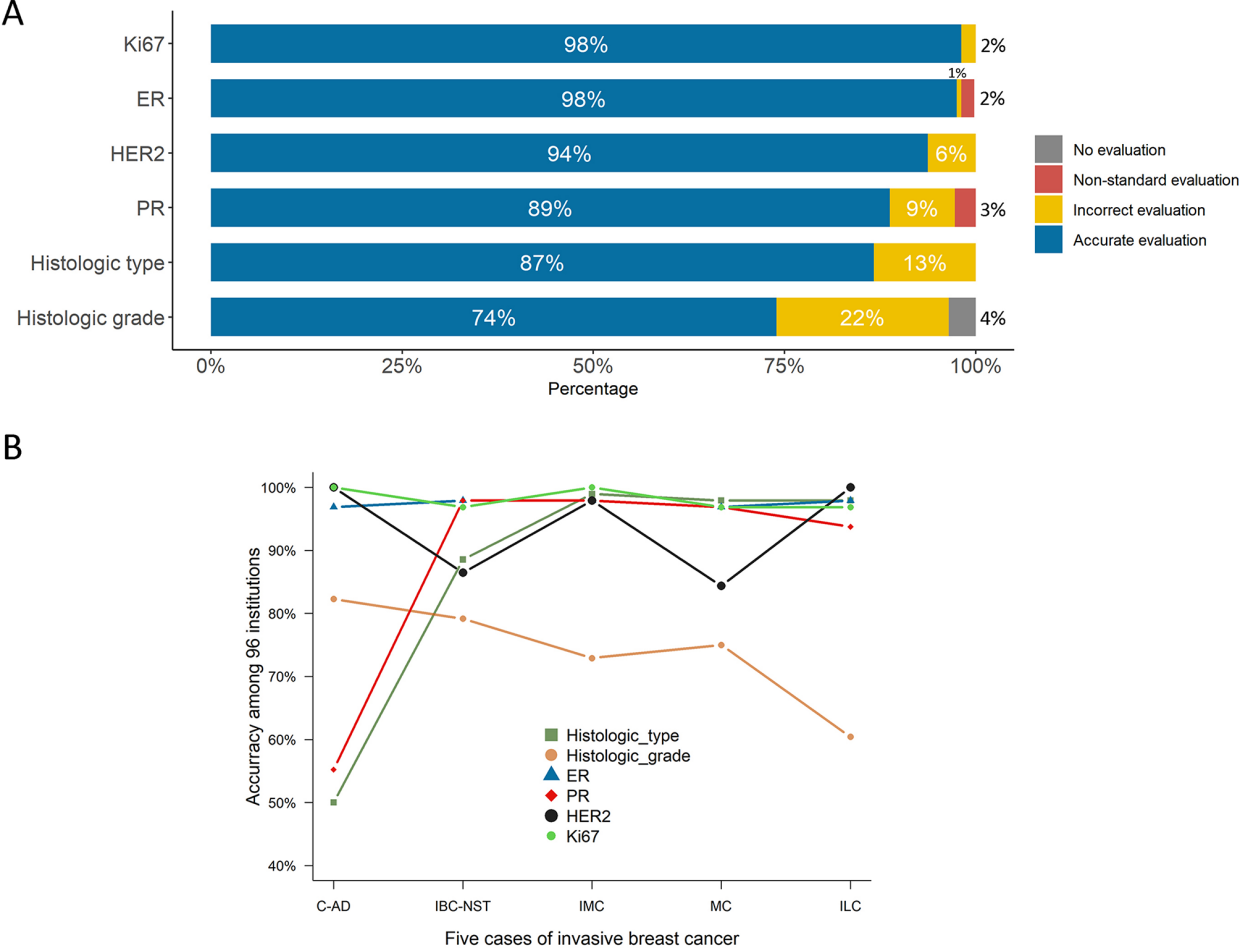
Overall performance of six key items. **A** Overall performance of six key items by 96 institutions. **B** Overall performance of six key items across each individual case

This PT program demonstrated satisfactory accuracy for histologic type (87%), ER (98%), PR (89%), HER2 (94%), and Ki67 (98%) (Fig. 4A). Regarding hormone receptors, ER and PR with non-standard assessments accounted for 2% and 3%, respectively (Fig. 3A). The histologic grade showed low accuracy (74.0%) (Fig. 4A), of which the inconsistent histologic grade accounted for 22%, and the grade not evaluated accounted for 4%.

Regarding specific cases determined by 96 institutions, C-AD showed low accuracy for histologic type (50%, 48/96) and PR (55%, 53/96). 45% (43/96) of the institutions incorrectly identified histologic type of C-AD as IBC-NST. Meanwhile, ILC demonstrated a relatively high proportion of incorrect histologic grades (40%, 38/96), including incorrect evaluation (34.4%, 33/96) and no evaluation (5.2%,5/96) (Fig. 4B).

As to the interobserver variability of six key items among five cases, the results from participants showed perfect interobserver agreement for ER (ICC = 0.947) and PR (ICC = 0.916). It exhibited good interobserver agreement for Ki67 (ICC = 0.646) and histologic type (κ = 0.799), and fair agreement for histologic grade (κ = 0.274).

### Overall analysis of unacceptable results

Unacceptable results can be separated into three categories: incorrect evaluation (86.7%), non-standard evaluation (7.5%), and no evaluation (5.8%) (Table 2).

**Table 2:** Overall analysis of unacceptable results

Overall analysis of unacceptable results (100%)	
**Incorrect evaluation (86.7%)**	
Incorrect identification of C-AD as IBC-NST	15.7%
Incorrect evaluation of histologic grade	36.7%
Incorrect evaluation of ER/PR/HER2/Ki67	28.2%
Others	6.1%
**Non-standard evaluation (7.5%)**	
Inappropriate use of 1+/2+/3 + rather than staining percentage for ER/PR	6.1%
Misclassification of ER/PR < 1% weak expression as positive staining	1.4%
**No evaluation of histologic grade in ILC, MC and IMC (5.8%)**	

Among the causes of incorrect evaluation, inconsistent histologic grade accounted for 36.7%; incorrect identification of C-AD as IBC-NST accounted for 15.7%; and inaccurate evaluation of ER/PR/HER2/Ki67 accounted for 28.2% (Table 2). Among the causes of non-standardized evaluations, some were inappropriate use of 1+/2+/3+ rather than the staining percentage of cancer cells for ER/PR (6.1%); and some were misclassification of ER/PR < 1% weak expression as positive staining (1.4%) (Table 2). Moreover, no evaluation of histologic grade in ILC, MC, and IMC was found, accounting for 5.8% (Table 2).

### Distribution of PT scores across 96 participating institutions

In this study, the average and median PT scores were 87.8 and 89.5, respectively. The maximum and minimum scores were 100 and 54, respectively.

**Fig. 5:**
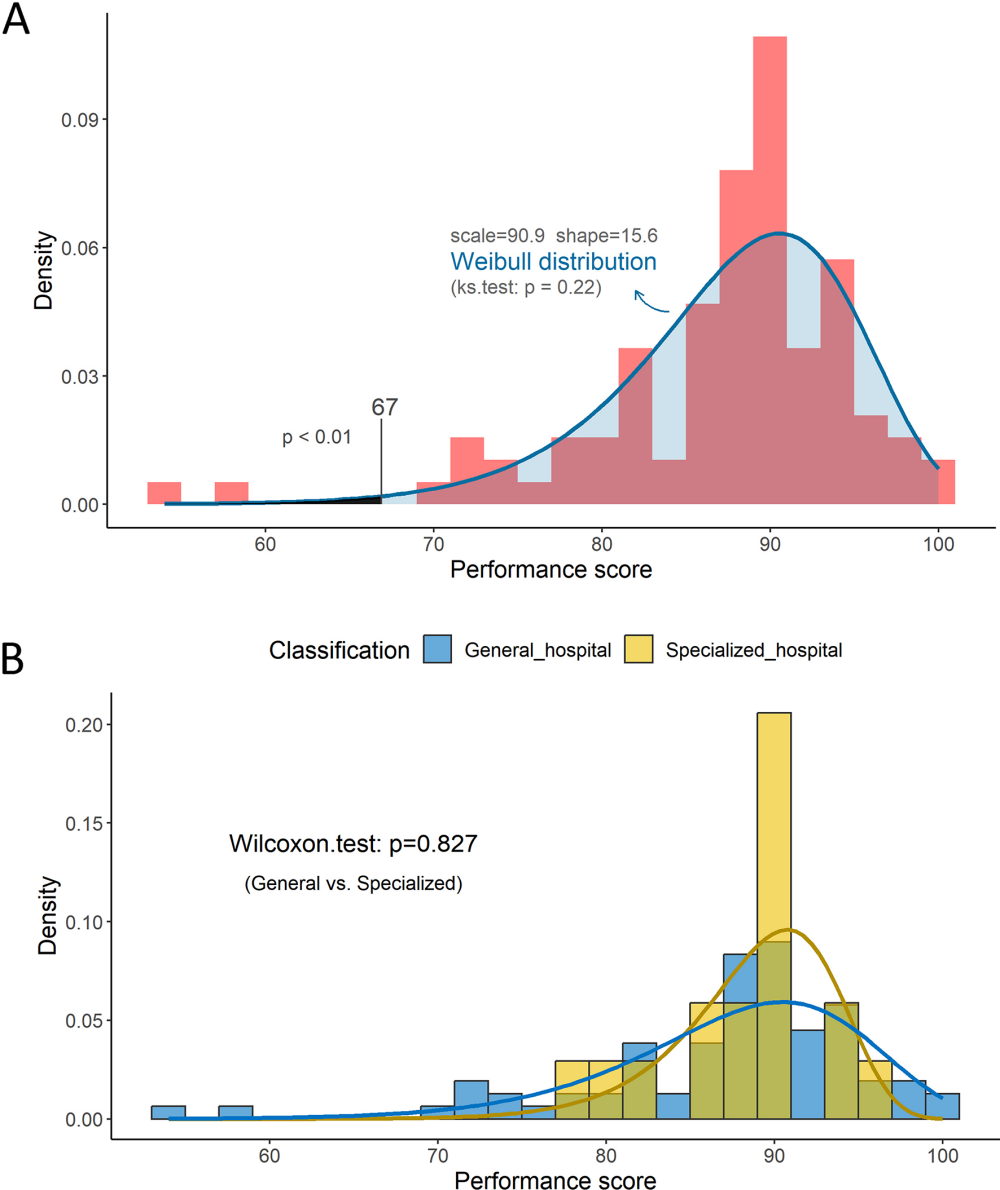
Overall distribution of PT scores. **A** Overall distribution of PT scores across 96 institutions. **B** Distribution of PT scores between general and specialized hospitals (b) were exhibited

As expected, the PT scores across the 96 participating institutions demonstrated a left-skewed distribution, which fitted well using the Weibull distribution. The scale and shape values were 90.9 and 15.6, respectively. KS.test for the Weibull distribution yielded p = 0.22. Based on probability, a score below 67 indicated p < 0.01. Thus, the cut-off score for unacceptable PT performance was 67. Accordingly, two participating institutions with scores below 67 were deemed unacceptable and required further improvement (Fig. 5A). Meanwhile, there was no significant difference in PT score between general and specialized hospitals (p = 0.827) (Fig. 5B).

## Discussion

The quality of pathology reports is vital for cancer care and monitoring. A thorough and accurate report can assist clinicians in selecting the most appropriate treatment for each patient. Pathologists are currently expected to be aware of both domestic and international guidelines for breast cancer diagnosis, but it is still unclear how successfully these guidelines have been integrated into routine clinical practice in China. Thus, in 2022, NCC/NQCCC and CNAS collaborated to launch this national PT scheme for the Pathological Diagnostic Capability of Breast Cancer in China, which is pivotal to the baseline assessment of diagnostic capability for pathologists, and also crucial for quality control and future improvement.

In this PT program, six key pathological items for breast cancer were chosen to evaluate the performance of pathological diagnostic capability of the participants. These items impacted not only the accurate pathological diagnosis, but also the therapeutic decision and prognostic indication. Therefore, these items was recommended for mandatory evaluation in all invasive breast carcinoma [9, 11–13], which were also strongly recommended as essential quality indicators in breast cancer care by EUSOMA [14, 15].

Our study showed high performance of IHC evaluation, including ER, PR, HER2, and Ki67, but relatively low accuracy of histological assessment of tumor type and grade. The most obvious example is that 45% (43/96) of institutions incorrectly determined the histologic type of C-AD as IBC-NST. This indicates that some institutions mainly focus on the interpretation of IHC, while paying less attention to morphological changes, which is a matter of some concern. There may be several reasons for this phenomenon. First, according to the current NCCN guideline, many subtypes of invasive carcinoma are treated similarly, and the therapy selection may largely depend on the IHC findings of ER, PR, and HER2 [13]. Second, China now has well-established EQA program at a national level to assess the consistency and accuracy of IHC staining and the interpretation of ER, PR, and Ki-67 [16]. However, before the implementation of our PT program, a thorough nationwide assessment of pathologists’ diagnostic skill was still lacking in China, Therefore, it seems that this phenomenon might be a systemic issue at present, which require further investigation. While, a fact we need to highlight is that the NCCN treatment recommended therapeutic algorithms still begin with histologic subtype, followed by the ER, PR, and HER2 status. Some of the pure subtypes of breast cancer, such as mucinous carcinoma as mentioned in the NCCN, are thought to have better outcomes than others, and are treated differently [13]. Even for the C-AD, the currently inconsistent findings of clinical follow-up may be partial due to the use of different criteria for the diagnosis, as indicated by the latest WHO classification [9]. Thus, it is necessary to adhere to the strict diagnostic criteria for the diagnosis of histologic types, at least it would be helpful to identify the pure histologic type or a combination with other subtypes, which, in turn, facilitates future clinical research. Therefore, to better improve the overall capability of pathological diagnosis of breast cancer in China, training and education in the related guidelines and recommendations, making them more explicit, are still necessary.

Furthermore, our study showed 34.4% (33/96) of institutions having the wrong histologic grade. The interobserver agreement for histologic grade among participants in our investigation (κ = 0.274) was relatively lower than that observed in PS Ginter et al. study which indicated a moderate level of agreement using digital slides (κ = 0.497) [17]. This discrepancy could be partially due to the lack of consensus or guideline for grading tumors on digital slides. Meanwhile, interobserver agreement for histologic type among participants in our study (κ = 0.799) was higher that of external quality assurance scheme in UK (κ = 0.57) [4], and a study involving 12 different countries in Europe (κ = 0.58) [5]. This difference was likely due to the limited subtypes of cancer for testing in our study, which required further improvement.

Meanwhile, regarding non-standard evaluation or even not carrying out evaluation for some key items, the template-based synoptic reports/checklist pathological reporting system would be able to effectively address these issues. Template-based synoptic reporting for breast resection specimens has been shown to improve the comprehensiveness of pathology reports and clarity of data organization, generating a more user-friendly report for clinicians [18]. Despite the widespread recognition of the efficacy of synoptic reporting, its implementation in China remains limited. Our institution developed a template-based synoptic report system in 2021 for surgical resection of breast cancer specimens, which significantly improved the reporting quality and variability among pathologists. Given the problems highlighted by this national PT scheme, we will share our center’s experiences in the near future to improve the inter-laboratory variability and comprehensiveness of pathology reports, such as the construction of a tailor-made template-based synoptic report system and its integration into the currently used traditional narrative descriptive reporting system.

There are some limitations to our study. First, although the integration of digital slides into routine clinical practice is increasingly, evaluating pathologic changes on digital slides is still a challenge for many pathologists. Second, despite offering the standardized software (KF-Viewer) for participants, we did not establish standardized requirements for the setting of the display and other hardware components, which may introduce potential bias for the testing. Third, the number of testing cases in our schemes is five, which seems to be relatively small.

In order to addressed the abovementioned shortcomings, several actions can be taken in future rounds of the PT program. Firstly, training for interpretating pathologic changes on digital slides is necessary, especially the instruction on tumor grading. Secondly, conducting a survey of the participants in learning about their daily-used hardware configuration, particularly the monitor. Accordingly, we try to setup a widely acceptable and accessible criteria for the display and other hardware components. Thirdly, the number and subtype of cases for testing should be expanded to acquire more comprehensive baseline assessment of the diagnostic capability of pathologists in China. Last but not the least, since the promising clinical use of Trastuzumab deruxtecan (T-DXd) in patients with IHC-based HER2-Low (1+, and 2+/FISH−) metastatic breast cancer was revealed by the Destiny-Breast04 study [19], the identification of a subset of “HER2-Low” breast cancers has attracted pathologists attention. Thus, in the next round of PT scheme, besides including the full spectrum of grades of HER2-IHC in the testing cases, we also intend to conduct a more comprehensive investigation into the HER2-low interpretation in China, which may benefit from upcoming more T-DXd-like HER2-targeted therapies.

Moreover, this nationwide PT program was a pilot study that enrolled only the first half of the members of the NSDQCP for breast cancer. In 2023, we will launch the next round of scheme to evaluate more member institutions; thus, we will acquire a more comprehensive view of the performance of pathologists in China. We believe that this PT scheme will play a crucial role in constantly monitoring and improving the competence of Chinese pathologists in breast pathology.

In conclusion, our study exhibited a satisfactory baseline assessment of the diagnostic capability of pathologists in China. More importantly, we identified several problems that could be improved by clarifying related pathology guidelines and implementing a template-based synoptic reporting system in the future.

## Data Availability

The data of this study are available from the corresponding author J.Y. (email: jmying@cicams.ac.cn), upon request.
